# The Role of Rehabilitation in the Management of Patients with Charcot-Marie-Tooth Disease: Report of Two Cases

**DOI:** 10.3889/oamjms.2016.079

**Published:** 2016-07-12

**Authors:** Erieta Nikolikj Dimitrova, Ivana Božinovikj, Simona Ristovska, Aleksandra Hadzieva Pejcikj, Aleksandra Kolevska, Mirjeta Hasani

**Affiliations:** *Institute of Physical Medicine and Rehabilitation, Medical Faculty, Ss Cyril and Methodius University of Skopje, Skopje, Republic of Macedonia*

**Keywords:** Charcot-Marie-Tooth disease, rehabilitation, exercise, orthoses

## Abstract

**BACKGROUND:**

Charcot-Marie-Tooth (CMT) disease is a hereditary disease with signs of chronic non-progressive motor-sensory neuropathy which is characterised by symmetric muscle atrophy and weakness of the distal portion of lower extremities.

**AIM:**

The aim is to present two cases with peroneal muscular atrophy, applied rehabilitation procedures and rehabilitation outcome.

**MATERIAL AND METHODS:**

Patient DR, aged 51, and patient KH, aged 78. Both patients had weakness and pronounced atrophy of the distal portion of lower extremities, numbness down the legs, contractures in the ankles and walking difficulties. Evaluation of patients included a clinical examination, Barthel Index, Time Up and Go test, measurement of the ankle range of motion, and a manual muscle test. On admission, the Barthel Index score was 60 in the first case, and 80 in the second. The rehabilitation program included exercise therapy with for lower extremity, occupational therapy, stationary bicycle riding, galvanic current, water exercises, and ankle-foot orthoses for both legs.

**RESULTS:**

The therapy applied had no significant changes in the clinical neurological status of the patients, but yet it provided some improvement in ankle contractures, better mobility, and a more stable gait.

**CONCLUSION:**

The application of rehabilitation procedures in patients with Charcot-Marie-Tooth disease can improve their functional status and walking stability.

## Introduction

Charcot-Marie-Tooth (CMT) disease is the most common rare inherited neurological disorder with prevalence 1:2500. It represents a genetically heterogeneous group of symmetrical peripheral motor-sensory neuropathies sharing the same clinical phenotype characterised by distal limb muscle wasting and weakness, skeletal deformities, distal sensory loss and reduced deep tendon reflex [1], pes cavus or pes cavovarus, functional ankle instability and higher falls frequency [2]. The most frequent are demyelinating form (CMT 1) that is a result of the chronic progressive axonal degeneration and reinnervation with segmental demyelinisation; and axonal form (CMT 2) has electrodiagnostic findings more consistent with axonal loss involving the anterior horn cell of the spinal cord without evidence of segmental demyelination [3, 4]. They account for over 70% of cases [4].

These disorders are characterised by slowly progressive neuropathic processes involving the distal muscles with a highly variable severity and onset. Foot drop or steppage gate, foot pain secondary to foot deformity is also common [4]. The disease gradually progresses causing smaller or greater disability. Occasionally, there is also a weakness in the distal parts of the upper extremities.

Concerning the therapies, there is no pharmacology treatment [5]. The treatment procedures of such patients include rehabilitation, as well. Since muscle wasting and sensory disturbance are the main features of these syndromes, treatments aim to improve motor impairment and sensory disturbances [3] The rehabilitation processes are directed toward the prevention of contractures, maintenance of circulation, strengthening of the remaining unaffected muscles, improvement of gait, and improvement of quality of life [4, 6].

The aim is to present two patients with Charcot-Marie-Tooth (CMT) disease and the effects of the applied rehabilitation procedures.

## Material and Methods

*Design architecture:* The proposed study was a report of two cases. The report was interventional with subjects acting as their own control. The comparison was made between the pre and post outcome measures. The follow up was made 3 months later.

*Outcome measures:* The outcome measures in this study included: clinical examination, measurement of active and passive ankle range of motion with goniometer, force capabilities were tested for dorsal and plantar flexor muscles according to the Medical Research Council 0-5 Scale (Manual Muscle Testing) by same clinician, functional parameters with Time Up and Go test (three timed tests of motor function were assessed to stand from the chair and walk 3 meters, turn on back, go back and sit on the chair) (7), 6 meters walking test (three timed tests of motor function were assessed including time taken to walk 6 meters at the participant’s fastest and self-selected speed), Barthel Index for assessing disability, Visual Analogue Scale (VAS) for low back pain.

## Cases Presentation

### Case 1

Patient DR, aged 51. On admission, the patient complained of weakness in his extremities, more pronounced in lower parts, numbness down his arms and legs, occasional pain in lower back and in the back of his right hip. The disease started 4 years ago with weakness in the distal parts of extremities, more pronounced in lower extremities, with pains in his legs, walking difficulties, and fatigue. The following year, the patient was hospitalised at the Department of Neurology, where he was diagnosed with Charcot- Marie-Tooth type II disease.

EMG of chronic neurogenic lesions of moderate to a severe degree, mainly in the distal limb muscles with combined characteristics, mainly axonal damage. Computed tomography of the brain showed normal findings. EEG: on the basis of proper brain activity, groups of high-volt and slow waves were registered bilaterally frontotemporal.

Neuropsychological testing: the behavioural plan is dominated by depressive symptoms; the person is inhibited, hypersensitive, emotionally immature, and registers the phenomenon of fear and insecurity leading to a chronic anxiety condition.

Patient environmental and social history: finished primary school, unemployed, the beneficiary of social assistance, unmarried, lives with his parents, suffers from epilepsy, and receives medical treatment with the table. Carbamazepine a 200 mg 2 x 1, table. Phenobarbital a 100 mg 1 x 1.

#### Clinical examination on admission

Normal posture of the spine, no tension in paravertebral muscles in the cervical and lumbar area. Neck movements are partly limited with pronounced latero flexion and rotation; movements in the lumbar area are easily restricted in all directions.

*Upper Extremities:* postural tremor of fingers. Muscles with relatively preserved trophic. Active movements performed in all the joints to its full extent. Reduced rough motion force of both hands was noticed. Tendon reflexes give numerous bad responses, almost extinct. Superficial sensibility shows hand hyperesthesia. He uses his arms in everyday life.

*Lower Extremities:* both feet have equinovalgus deformity, with thumbs flexed. There is a pronounced weakness of the below knee muscles, more pronounced in the left leg. The muscles of the calves with reduced tone. The patient maintained their posture by balancing. He performed active movements of the hips and knees in quite a limited extent, in the ankles plantar flexion on both sides is 10 degrees, actively runs dorsal flexion in the partial volume due to muscle weakness (MMT 2), and passively it is minus 5 degrees to the left and 0 degrees to the right. Patellar reflex with the bilaterally symmetric response, Achilles tendon reflex is extinguished on both sides. He has somewhat reduced sensitivity to touch of the knees down, more pronounced in his left leg. He stands on his toes, but cannot stand on his heels. His gait is without aids, a typical peroneal gait, on a large scale.

Barthel Index score was 60 on admission. Time Up and Go test 11.7 sec. VAS for lumbal pain was 4. Walking time at a distance of 6 meters was 9.02 sec.

**Figure 1:**
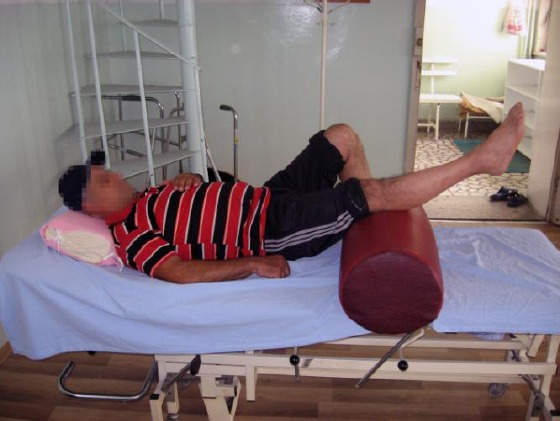
Active exercises for quadriceps

Following procedures were applied during rehabilitation: exercise therapy with active exercises for hip and knee muscles (Fig. 1), passive exercises for foot muscles, stationary bike ride, occupational therapy, manual massage and galvanization of lower extremities. Infrared rays and diadynamic current (DF, CP, LP 6 minutes) were applied for low back pain. Two ankle-foot orthoses for both legs to limit ankle plantar flexion were prescribed.

Upon dismissal, the clinical findings of the patient indicated no significant improvement in the lower leg muscle strength, no changes in MMT, a slight improvement of the left ankle contracture, and dorsal flexion passive 0 degrees. The patient’s gait was more stable with two ankle-foot orthoses. Barthel Index scores 60, Timed Up and Go test 10.3 sec. Walking time at a distance of 6 m. was 8.64 sec. The low back pain has disappeared - VAS was 0.

Follow-up examinations after 3 months have shown that the clinical findings and the Barthel Index are the same as on discharge. The patient continued to exercise at home.

### Case 2

Patient KH, aged 78. On admission, the patient complained of weakness in his lower extremities, numbness down legs, swelling and pain in his left ankle, difficulties when walking. The disease started some 38 years ago showing weakness in the distal portions of his lower extremities. He was examined by a neurologist in the regional hospital and diagnosed with Atrofia neural progressive. The muscle weakness was gradually developing 35 years ago; the patient was hospitalised at the University Department of Neurology, where the tests confirmed the diagnosis of Charcot-Marie-Tooth disease.

EEG in favour of deficit dominant in the muscles of the lower extremities most pronounced in the small toe, and to a lesser extent at the m. tibialis and m. quadriceps femoris. There is a large reduction in motor units, while the remaining motor units show signs of hypertrophy. Findings of the upper extremities are within limited values. There is a reduced conduction n. peroneus and a great loss of motor axons. The upper extremity findings are in favour of segmental demyelination.

The patient has been repeatedly subjected to spa-balneotherapy. He was admitted to our rehabilitation institution for the first time. Patient environmental and social history: completed higher education, retired, worked as a school teacher, suffers from arterial hypertension.

#### Clinical examination on admission

*Upper Extremities:* increased contours of the PIP and DIP joints on both hands are present; more pronounced in the DIP joints. Hand muscules have a slight hypertrophy corresponding to his age. The patient fully performs active movements in all of his joints. Superficial sensibility is normal. Tendon reflexes have weak response levels. Rough motion force of both hands is slightly reduced. He uses his hands in everyday life.

*Lower Extremities:* Both feet are in the equine position. There is a small swelling in the left ankle, painless to palpation. The lower limbs have a pronounced hypotrophy and muscle hypotonia. The patient maintains his lower extremities in posture. He performs active movements in both hips and knees to the full extent, slightly slower, whereas his active movements are possible in ankles in traces only because of the pronounced muscle weakness (MMT 1+), plantar flexion is mutually 10 degrees, active dorsal flexion is possible in traces minus 5 degrees left and minus 10 degrees to the right, and passive dorsal flexion is 0 st. Superficial sensibility is preserved. Tendon reflexes are extinguished. He stands on his toes, but not on his heels. He walks without walking aids, with a typical peroneal gait on both sides.

Barthel Index scores 80 on admission. Time Up and Go Test 13.54 sec. Walking time at a distance of 6 meters was 9.99 sec. VAS for pain in his left ankle was 4.

**Figure 2:**
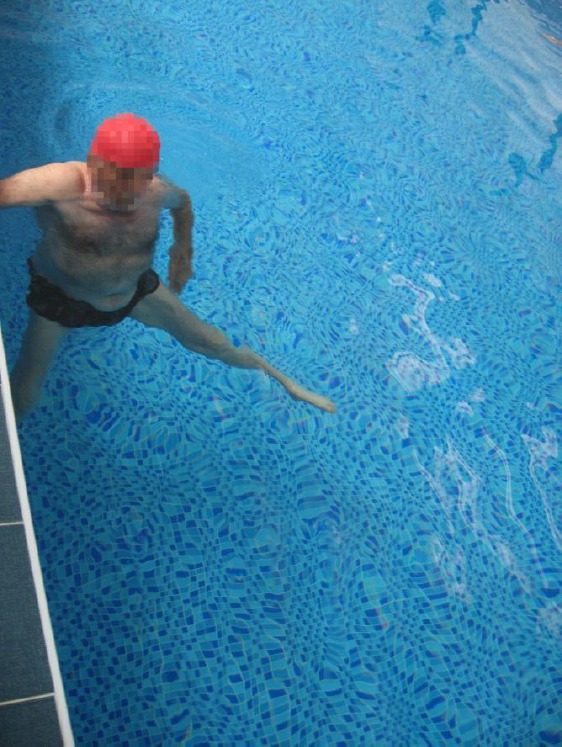
Water exercises for lower extremities in the pool

Following procedures were applied during rehabilitation: exercise therapy with active exercises for hip and knee muscles, passive exercises for foot muscles, stationary bicycle riding, and water exercises in the pool (Fig. 2), occupational therapy, manual massage and galvanization of lower extremities. Dyadinamic currents (DF, CP, LP 6 minutes) were applied for swelling and slight pain in the left ankle. Two ankle-foot orthoses for both legs to limit ankle plantar flexion were prescribed.

Upon dismissal, the patient had subjective improvement, but the clinical findings of the patient indicated no significant improvement in the lower leg muscle strength, no changes in MMT, swelling decreased in the left ankle, no pain in the left ankle. The patient’s gait was more stable with below-knee plastic orthoses. Barthel Index scores 80, Timed Up and Go test 12.3 sec. Walking time at a distance of 6 m. was 9.14 sec.

Follow-up examinations after 3 months have shown that the clinical findings and the Barthel Index are the same as on discharge. The patient continued to exercise at home.

## Discussion

There are no effective drugs for therapeutic approach to Charcot-Marie-Tooth (CMT) disease at present [8]. Six RCTs compares the effect of oral ascorbic acid (1 to 4 grammes) and placebo treatment in CMT 1A. High-quality evidence indicates that ascorbic acid does not improve the course of CMT1A and outcomes in adults, as well as by low-quality evidence in children [5].

Treatments are aimed at functional restoration of gait with an orthosis, exercises and surgery [9], and improvements in sensory disturbances and pain reduction [3, 9]. Rehabilitation is usually suggested by physicians, but only a few studies were performed in this field and some evidence confirmed the benefit deriving from physical therapy. The rehabilitation programs are heterogeneous, differ from therapist to therapist, should be tailored to the patients and may be changed from time to time. Moreover, usually, the patient is not able to define and quantify precisely the type of rehabilitation procedures [8]. It is a rare disease with published studies with case reports, or studies with small numbers of patients and that is why there is no high-quality evidence for suggested methods of rehabilitation.

Carter GT, et al. believe that patients with Charcot-Marie-Tooth disease should be treated in a comprehensive multi-disciplinary ward in which a neurologist, physiatrist, orthopaedic surgeon, physiotherapist, occupational therapist and an orthopedist should be involved. Treatment should be aimed at achieving maximum independence and quality of life [6].

In an observational study of total 26 adult patients with CMT, the results suggested that gait parameters correlated with both dorsal- and plantar – flexors strength, whereas postural parameters correlated only with plantar-flexor strength. They concluded that improved knowledge of postural and gait capacities may constitute a basis to emphasise the corrections that should be enabled by rehabilitation exercises or orthotic devices [10]. Rose et al. in the group of 29 children and adolescents participants with CME reported moderate to severe bilateral functional ankle instability, reporting functional ankle instability significantly associated with cavus foot structure, female gender and impaired balance [2].

Padua L, et al in the observational survey study with 123 patients with CMT, showed that patients have physical and mental benefit from rehabilitation, but also perceived that do not perform the best rehabilitation program for their pathology. They concluded that there was a lack of consensus on rehabilitation tailored to CMT patients need and familiar/caregiver [8].

During rehabilitation, our patients received Achilles tendon stretching exercises, ankle extension exercises, preserved muscles strengthening, occupational exercises, and water exercises in the pool for the second patient. The applied therapy enabled patients a certain reduction in ankle contracture, improved mobility, and more stable gait.

According to a systematic review, exercises may be beneficial to maintain strength and function for people with CMT. Significant effects described included improvements in strength, functional activities, and physiological adaptations the following exercise. There was few studies available and moderate quality of evidence. The optimal exercise modality and intensity for people with CMT as well as the long-term of exercise remain unclear [11]. Foot extension exercises are recommended to prevent shortening of the Achilles tendon [12].

Both patients received electrotherapy with galvanic current for both lower extremities, longitudinally because of its excitatory effect, as well as dyadinamic currents for the lumbar pain of the first patient and for the left ankle pain of the second patient. After 10 days of dyadinamic current application, the patients had no pain.

Some authors for rehabilitation of CMT patients recommended iontophoresis, electric stimulation (indirect and direct), dyadinamic currents (rhythm syncope), middle-frequency currents with alternative or constant regime with frequency from 100 to 150 Hz, depth of 100%, as well as exercises, exercises in water, four-cell bath, paraffin therapy, healing mud therapy and manual massage [13].

In the previously published case report a patient with CMT after rehabilitation treatment with galvanic currents, exercise therapy, occupational therapy, orthopaedic devices (ankle- foot orthosis and a pair of crutches) achieved some improvement in mobility and gait [14].

On admission our patients walked with a typical peroneal gait, without walking aids, whereas on discharge, their gait was with two ankle-foot orthoses for limiting the ankle plantar flexion without walking aids. The mobility and gait were better, more stable then on admission.

Special shoes with good ankles support may be necessary. Good evaluation of movements, application of ankle foot orthosis and training for its application is indispensable. Patients frequently need AFO orthosis for foot equines correction and gait improvement [12]. In the study of 7 Charcot-Marie-Tooth type 1A patients, the use of anterior elastic ankle-foot orthoses improved walking economy by reducing the energy cost of walking per unit of distance, thus reflected a lower level of metabolic effort and improved mechanical efficiency in comparison with shoes only [15].

Some patients need under-elbow crutches or a stick to improving their gait stability, and only less than 5% of the patients need a wheelchair. It is desirable to educate the patients in relation to weight control because obesity makes difficulties in movement. Exercises should support the capacity of each individual patient. The majority of the Charcot-Marie-Tooth patients remain physically active [16]. Adapted aids may be used occasionally to improve the activities of daily living and self-care. Wrist and hand orthoses are occasionally used. Professional occupational therapy important for employment and career may also be needed [16].

In a clinical study testing sensitivity of various scales in rehabilitation and lungs condition with 8 CMT patients compared to healthy persons, it was determined that all the rehabilitation measures were much worse with CMT patients than was the case with the healthy persons. Treatments with walking belts, extension exercises, respiratory exercises and proprioceptive exercises were carried out twice a week for a period of 8 weeks. After the treatment, there was a significant improvement in the movement range of the ankles and in the walking time at a distance of 6 meters. The authors concluded that patients should be treated at least 2 times a year because regression of outcome measures was determined after a 6-month period without exercises [17].

In the recently published survey of 44 patients with CMT disease, the qualitative content analysis revealed that personal factors such as fatigue, poor balance, muscle weakness, and pain were important barriers to physical activity behaviour. They concluded that understanding of these factors can guide health professionals to facilitate physical activity behaviour in this group of patients [18].

According to the systematic review larger randomised controlled trials are needed for any form of pharmacological intervention as well as for the form of physical intervention [3].

In conclusion, the application of rehabilitation procedures in patients with Charcot-Marie-Tooth disease can improve both their functional status and walking stability. Treatment decisions must be individualised and based upon a clear history, careful examination, and well-defined patient goals.
